# Modeling and Validation of Fatigue and Recovery of Muscles for Manual Demolition Tasks

**DOI:** 10.3390/ijerph19020930

**Published:** 2022-01-14

**Authors:** Cannan Yi, Fan Tang, Kai-Way Li, Hong Hu, Huali Zuo, Caijun Zhao

**Affiliations:** 1 School of Safety and Management Engineering, Hunan Institute of Technology, Hengyang 421102, China; cannanyi@hnit.edu.cn (C.Y.); honghu@hnit.edu.cn (H.H.); Hualizuo@hnit.edu.cn (H.Z.); d10803010@chu.edu.tw (C.Z.); 2 School of Mechanical Engineering, Southeast University, Nanjing 211189, China; 3 Department of Industrial Management, Chung Hua University, Hsinchu 30012, Taiwan

**Keywords:** manual demolition task, muscle fatigue, muscle fatigue recovery, prediction modeling, work-related musculoskeletal disorders (WMSDs)

## Abstract

Manual demolition tasks are heavy, physically demanding tasks that could cause muscle fatigue accumulation and lead to work-related musculoskeletal disorders (WMSDs). Fatigue and recovery models of muscles are essential in understanding the accumulation and the reduction in muscle fatigue for forceful exertion tasks. This study aims to explore the onset of muscle fatigue under different work/rest arrangements during manual demolition tasks and the offset of fatigue over time after the tasks were performed. An experiment, including a muscle fatigue test and a muscle fatigue recovery test, was performed. Seventeen male adults without experience in demolition hammer operation were recruited as human participants. Two demolition hammers (large and small) were adopted. The push force was either 20 or 40 N. The posture mimicked that of a demolition task on a wall. In the muscle fatigue test, the muscle strength (*MS*) before and after the demolition task, maximum endurance time (MET), and the Borg category-ratio-10 (CR-10) ratings of perceived exertion after the demolition task were measured. In the muscle fatigue recovery test, *MS* and CR-10 at times 1, 2, 3, 4, 5, and 6 min were recorded. Statistical analyses were performed to explore the influence of push force and the weight of the tool on *MS*, MET, and CR-10. Both muscle fatigue models and muscle fatigue recovery models were established and validated. The results showed that push force affected MET significantly (*p* < 0.05). The weight of the tool was significant (*p* < 0.05) only on the CR-10 rating after the first pull. During the muscle fatigue recovery test, the *MS* increase and the CR-10 decrease were both significant (*p* < 0.05) after one or more breaks. Models of MET and *MS* prediction were established to assess muscle fatigue recovery, respectively. The absolute (AD) and relative (RD) deviations of the MET model were 1.83 (±1.94) min and 34.80 (±31.48)%, respectively. The AD and RD of the *MS* model were 1.39 (±0.81) N and 1.9 (±1.2)%, respectively. These models are capable of predicting the progress and recovery of muscle fatigue, respectively, and may be adopted in work/rest arrangements for novice workers performing demolition tasks.

## 1. Introduction

Work-related musculoskeletal disorders (WMSDs) are disorders of muscles, bones, nerves, tendons, ligaments, joints, and cartilages in different body segments caused by factors, such as excessive load, vibration, repetitive operation, and awkward posture [1]. They have become the second most common occupational disease in many developing and industrialized countries. Many countries, such as the United States, Britain, Germany, and Japan, have listed these disorders as occupational diseases [2]. In China, the literature has shown WMSDs are common at work in hospitals [3], coal mines [4], factories [5], farms [6], transportation [7], and construction sites [8]. Although hand–arm vibration syndromes have been included as one of the occupational diseases in China [9], other WMSDs, such as low-back pain and neck–shoulder syndrome, are still not included even though the workers are suffering. Prevention of WMSDs is one of the primary missions in the communities of occupational safety and health.

Muscle fatigue is common at work. Frequent muscle fatigue is an early symptom of WMSDs [10,11,12]. Muscle fatigue may be due to the length of work time, excessive load, awkward posture, or insufficient rest time [12,13,14]. Recovery of muscle fatigue refers to the functional capacity regrowth of body tissues after the onset of fatigue [15]. Investigating the mechanisms and patterns of the development of muscle fatigue and recovery during rest is helpful in providing a theoretical basis from which to reduce the onset of muscle fatigue and thus reduce the risk of WMSDs for workers. Modeling the process of muscle fatigue and recovery provides an opportunity to describe the effects of time of work, workload, and other task parameters on muscle fatigue and recovery. Muscle fatigue may be indicated by a decline in muscle strength (*MS*) after a period of forceful exertion. Changes in the maximum *MS* and maximum endurance time (MET) have been widely adopted in the modeling of muscle fatigue [12,15,16,17,18]. For both *MS* and MET, both experimentally-based and theoretically-based models have been reported [15,19,20]. The former was normally constructed by asking the participants to perform specific tasks using certain postures. Those models, apparently, are valid only to the tasks and postures that were tested. Some other models were, on the other hand, established considering the biomechanics of body segments which may not be task-specific. Recovery of muscle fatigue may be assessed by measuring the *MS* [21,22,23,24], heart rate [25], and surface electromyography of muscle groups [26,27,28].

Manual demolition tasks are performed when workers use hand-held tools to demolish existing structures. These tasks may involve drilling, cutting, and crushing. They are common in construction, municipal engineering, road and bridge engineering, and post-disaster demolition activities. They may lead to muscle fatigue and risk of WMSDs due to heavy load, awkward posture, length of work time, poor working environment, and tool vibration [29,30]. Existing research on muscle fatigue issues associated with manual demolition tasks have focused on vibration transmission [31,32], *MS* change [33] under different operating conditions. However, the development of muscle fatigue and progress of fatigue recovery for these tasks remain unclear.

The decline of muscle strength, subjective rating of muscle fatigue, and MET have been adopted to indicate the development of muscle fatigue [12,18,19,33]. This study aims to explore the development of muscle fatigue and the progress of muscle recovery for manual demolition tasks. The objectives were to determine the effects of the tool used and push force on the decrease in *MS*, subjective rating of muscle fatigue, and MET. In addition, predictive models were developed to quantify the asymmetric patterns of both the MET and *MS* for the manual demolition tasks. 

## 2. Materials and Methods

### 2.1. Experiment

An experiment was performed in the laboratory. The temperature was 23.9 (±1.1) °C, and the relative humidity was 55.6 (±11.3) %.

### 2.2. Apparatus and Tools

To simulate demolition tasks on a wall, a rig supporting a three-dimensional force sensor (FH3D-45, Nateen Technology Co., Ltd., Shenzhen, China) and a wooden target were installed. The force data of the force sensor were transmitted to a computer in a real-time manner. Two manual demolition hammers, a large one (GSH500, Bosch, Hangzhou, China) and a small one (GSB10RE, Bosch, Hangzhou, China), were purchased from a local hardware store. The lengths of these two hammers were 458 and 262 mm, respectively. The weights of them were 5.6 and 1.5 kg, respectively. A stopwatch was used to measure the *MET* and to monitor the time in the recovery test. In addition, a Borg category-ratio-10 (CR-10) scale [34] was adopted to measure the perceived ratings of exertion. 

### 2.3. Participants

**Figure 1:**
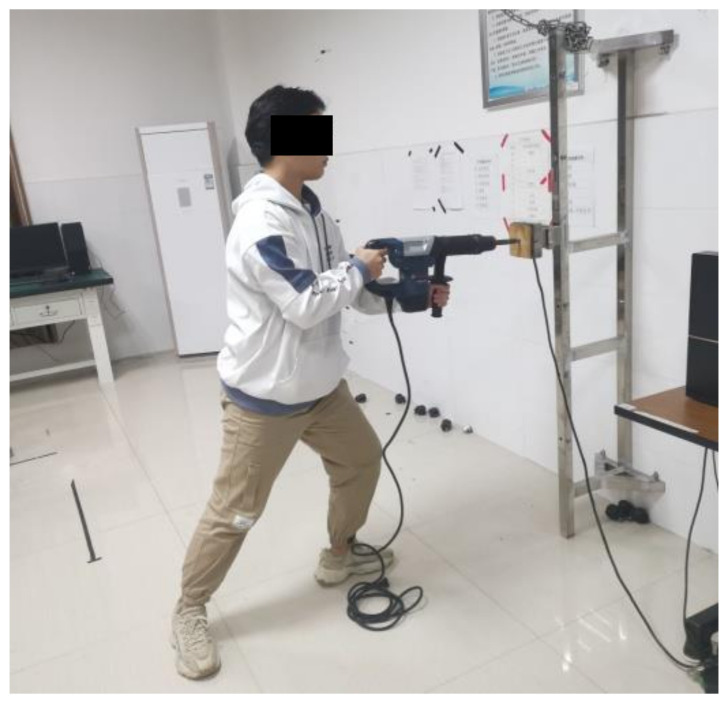
Demolition task in the experiment.

Seventeen male adults (19.35 ± 0.84 years; 171.32 ± 5.58 cm, 68.17 ± 10.54 kg, 23.16 ± 2.92 kg/m^2^) with no history of WMSDs within a year of the study were recruited. All had no prior experience in demolition hammer operation. This sample was a convenient sample. All of the participants were healthy and right-handed. Their shoulder height, elbow height, and knee height were 140.55 (±4.77) cm, 105.24 (±3.91) cm, and (50.84 ± 2.64) cm, respectively. These dimensions were measured using an anthropometer [35] when the participant was standing erect with the upper arm straight down and the lower arm at the horizontal. Before the experiment, the participants were informed about the purposes and procedure of the experiment. They read and signed the informed consent. Before the experiment, a professional demolition worker gave a brief onsite training of hammer usage on a target on a wall to all the participants. In the experiment, the participant held the hammer and pushed it onto a wooden target on a metal rig (see Figure 1) using the same posture as in their onsite training. The participants were encouraged to maintain this posture the best they could during the trial. 

### 2.4. Experimental Design

The literature indicates that muscle fatigue depends on the time and load of forceful exertion, while recovery from muscle fatigue depends primarily on time [12,23,36,37]. This experiment included tests of muscle fatigue and muscle fatigue recovery by measuring the MET, *MS*, and CR-10 scores [12,23,34,36,37]. 

In the muscle fatigue test, the participant pushed a demolition hammer against a target in front of them (see Figure 1) until he could not continue any longer. This mimicked a demolition task on a target from the front. The push force was either 20 or 40 N. These values were selected based upon the comments from local workers on a construction site in a pilot study. The push force activated a force sensor attached to the target. A computer monitor displayed the force in a real-time manner. It provided visual feedback on the force to the participant. In addition, an experimenter monitored the force on the monitor and provided verbal feedback to the participant. The participant adjusted his push force when the force applied deviated from the designated level [38]. The demolition target was 115 cm above floor level. This height is the average elbow height of the Chinese male population [39]. Both the MET and *MS* before and after the test were recorded. The *MS* before the test was the maximum voluntary contraction (*MVC*) [12,23,33,40], and the *MS* after the push was recorded as *MS*_0_. After the trial, the CR-10 was recorded based on Borg CR-10 [34] and was denoted as CR-10_0_.

In the muscle fatigue recovery test, participants rested for 6 min and their *MS* and CR-10 at the end of 1, 2, 3, 4, 5, and 6 min were measured. The *MS* and CR-10 of these measures were termed *MS*_i_ and CR-10_i_, respectively, for i = 1, 2,…, 6. 

### 2.5. Procedures

**Figure 2:**
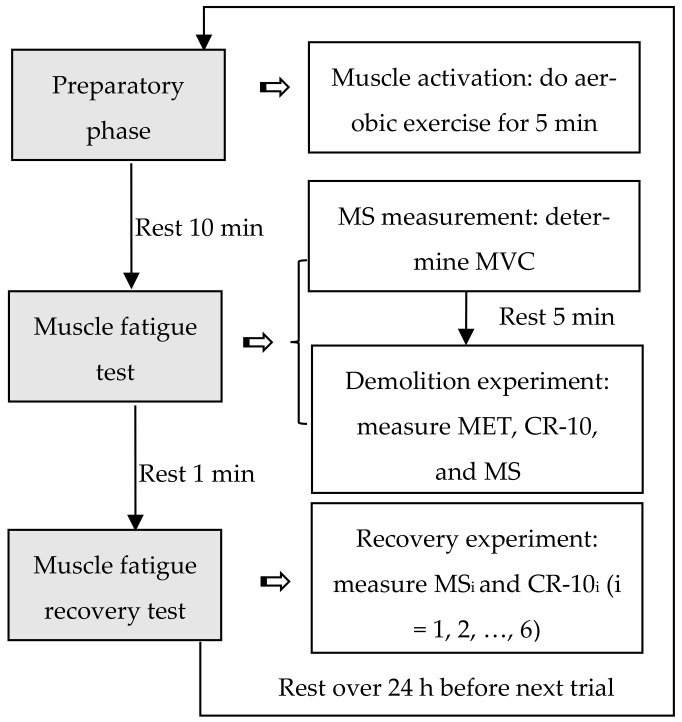
Experiment procedure: *MS*: muscle strength, *MVC*: maximum voluntary contraction, MET: maximum endurance time, CR-10: category-ratio-10.

Figure 2 shows the procedure of the experiment. At first, the participant completed a warmed-up exercise, following an aerobic fitness video from a commercial website [41] in the preparatory phase. After a rest of 10 min, he performed the muscle fatigue test. His *MVC* was measured and then the demolition experiment started. When measuring the *MVC*, the participant pushed the hammer with his maximum effort toward the target (see Figure 1) for approximately 5 s. The *MS* was measured three times [40] with an interval of 2 min between each measurement. The maximum reading of these three was recorded as the *MVC*. After the *MVC* measurement, the participant took a rest for 5 min and then performed the demolition trial. He held the hammer and pushed at an assigned force level under the guidance of an experimenter as long as he could. The time he could push was denoted as his MET. The participant reported his CR-10 after the push. This CR-10 was denoted as CR-10_0_. The end of a muscle fatigue test was the beginning of a fatigue recovery test. 

During the muscle fatigue recovery test, the participant took a rest and had his *MVC* and CR-10 measured every minute. To minimize the muscle fatigue accumulation of the participant during the recovery period, an experimenter took over the hammer. He placed the hammer in advance on the test position. The experimenter released and the participant took over the hammer and pushed. At the end of the test, the participant released the hammer, and the experimenter took it over and put it on the ground. Each participant needed to complete four trials; the factors were load or push force (20 N and 40 N) and tool (small, large). The order of the trial was arranged randomly.

### 2.6. Data Processing 

In the muscle fatigue test, a total of 68 MET data, 136 *MS* data, and 68 CR-10 data were recorded. In the muscle recovery test, a total of 408 *MS* and 408 CR-10 data were recorded. Descriptive statistics were conducted to show the MET, *MS*, and CR-10 scores under experiment conditions. Analysis of variance (ANOVA) was performed to determine the effects of hammer and load on the development of muscle fatigue and muscle fatigue recovery. The Bonferroni post hoc tests were conducted for posterior comparisons. Correlation analysis was carried out to show the relationship among measured parameters. Regression analyses were conducted to determine the MET and *MS* prediction models. Correlation coefficients were calculated to determine the relationships between the measured and predicted values. Microsoft^®^ Excel (Microsoft, Redmond, WA, USA) was used for preliminary data processing. The SAS^®^ 9.0 (SAS Institute Inc., Cary, NC, USA) was used for statistical analysis. A significance level of α = 0.05 was used.

## 3. Results

**Figure 3:**
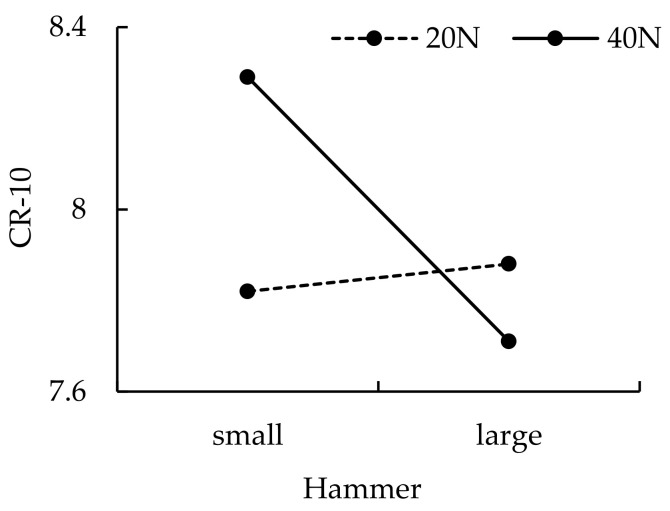
Interaction effects of push force and tool of CR-10_0_.

The MET values under experimental conditions are shown in Table 1. The load significantly (*p* < 0.0001) affected the MET. The Bonferroni post hoc test results showed that the MET of the 20 N load (7.43 ± 3.41) min was significantly higher than that of the 40 N condition (2.43 ± 0.73 min) (*p* < 0.0001). The tool significantly (*p* < 0.05) affected CR-10_0_. The Bonferroni post hoc test results showed that the CR-10_0_ of the small tool (8.06 ± 0.55) was significantly higher than that of the large one (7.79 ± 0.54) (*p* < 0.05). The effects of tool weight on MET, *MS*_i_, and CR-10_i_ (*i* = 1, 2, …, 6) were all insignificant. The interaction effect of push force and weight of tool on CR-10_0_ was statistically significant (*p* < 0.05) (see Figure 3). Pearson’s correlation coefficient *(r)* between the push force and MET was −0.72 (*p* < 0.0001).

A relative weight (RW) of the hammer versus the bodyweight of the participant was defined to further explore the effects of tool weight on the development of muscle fatigue. RW was in the range of 1.9% to 10.3%. It is possible to split participants into 3 groups: group L (1.9–4.7%), group N (4.8–7.5%), and group H (7.6–10.3). ANOVA was performed to analyze the effect of RW on MET. The result was not significant.

### 3.1. Modeling and Validation of Muscle Fatigue

The push force significantly affected MET (*p* < 0.0001), but tool weight was not significant on the MET. Tool weight was, therefore, not considered in the modeling of MET. The literature has shown that MET is a function of *f_MVC_ (f_MVC_* = load/*MVC*) [12,16,23,42]. Because *f*_MVC_ already contained load factors, load parameters were no longer introduced alone. The data were divided into groups A (20 N, small hammer; 40 N, large hammer) and B (20 N, large hammer; 40 N, small hammer). Data in group A were adopted in establishing the model, while group B data were used to validate the model. Correlation analysis results showed that the MET was negatively correlated (*r* = −0.73, *p* < 0.0001) with *f_MVC_*. The literature has recommended exponential and power function in MET modeling [12,16]. Equations (1) and (2) were adopted to incorporate these functions, respectively.

MET = k × e*^f^^MVC^*^ × c^ (1)

(2) $$\mathrm{MET}=k\;\times \;f_{MVC}^{c}$$

In Equations (1) and (2), both k and c are constant values. By logarithmic transformation of Equations (1) and (2), Equations (3) and (4) were obtained, respectively.

*L*n (MET) = *L*n (*k*) + c × *f_MVC_* (3)

*L*n (MET) = *L*n (*k*) + c × *L*n (*f_MVC_*) (4)

Linear regression analyses were conducted for Equations (3) and (4) where y is *L*n (MET) and x is *f_MVC_* and *L*n (*f_MVC_*) in Equations (3) and (4), respectively. The results are shown in Table 2. The model with the highest R^2^ (0.94), or y = −1.15x (Equation (d) in Table 2), was selected. This model was rewritten as Equation (5).

(5) $$\mathrm{MET}=f_{MVC}^{-1.15}\;$$

Validation of Equation (5) was performed. The absolute deviation (AD) and relative deviation (RD) calculated using Equations (6) and (7) were adopted to assess the fitness of the prediction models [18,42].

AD = |Predicted MET − Measured MET|(6)

RD = AD/Measured MET(7)

Model validation was performed by substituting group B data into Equation (5). The AD and RD using group B data were 1.83 (±1.94) min and 34.80 (±31.48) %, respectively. The AD and RD for group A data were 1.32 (±1.69) min and 30.49 (±27.22) %, respectively, and were slightly lower than that of group B. The MET model was, therefore, acceptable. 

When establishing MET models to assess muscle fatigue, a comparison with the models in the literature is usually conducted [12,16,40,42]. To further validate our MET model, we selected the MET models in the literature with posture or muscles involved similar to our tasks [12,16] and substituted group B data into them. The AD and RD are shown in Table 3.

Both the AD and RD of the current study using group A data were the lowest among all the models compared in Table 2. This implies that our MET model was appropriate in describing the MET-*f_MVC_* relationship in the manual demolition tasks tested in this study. For the other models in Table 2, the general model by Sjogaard [43] had relatively low AD and RD. The upper limb models of both Sato et al. [45] and Mathiassen and Ahsberg [46] and the back/hip model of Manenica [47] had relatively high RD values. This implies that the fitness of those model in predicting the MET of our demolition tasks were poor. 

**Figure 4:**
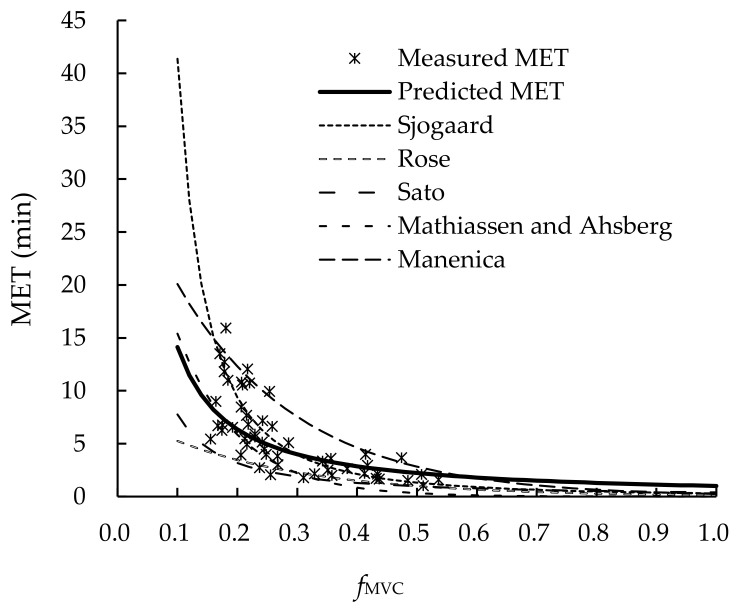
MET predictive models of muscle fatigue.

**Figure 5:**
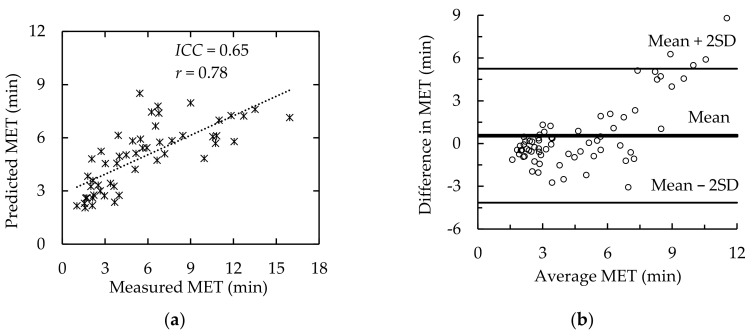
Comparison of measured and predicted MET. (**a**) *ICC* and *r*, (**b**) Bland–Altman agreement analysis. Difference in MET = Measured MET–Predicted MET; Average MET = (Measured MET + Predicted MET)/2.

The predicted MET using the models in Table 2 and the measured MET data are shown in Figure 4. When comparing data measured by different methods, both the intra-class correlation efficient (*ICC*) and Pearson correlation coefficient (*r*) are recommended [12,48]. In addition, Bland-Altman [49] recommended an agreement analysis for this purpose. Figure 5 shows the correlation and agreement between predicted MET using our model and all of our measured MET data. The *ICC* and *r* were 0.65 (*p* < 0.0001) and 0.78 (*p* < 0.0001) (see Figure 5a), respectively. Only a few data exceeded the Mean + 2SD upper bond (see Figure 5b). 

### 3.2. Modeling and Validation of Recovery of Muscle Fatigue

During the muscle fatigue recovery test, *MS* and CR-10 under four test conditions were insignificantly (*p* > 0.05) different. Therefore, we used group A data for modeling and group B data for validation. ANOVA was done to explore the effect of RW on the *MS*. It was found that RW was significant on *MS* at 0–6 min and CR-10 at 5 min (*p* < 0.05). The Bonferroni post hoc test showed that *MS* at 0–6 min and CR-10 at 5 min of group N was significantly (*p* < 0.05) higher than that of groups L and H. The difference between groups L and H was not significant.

During the muscle fatigue test, all participants stopped pushing because they could not push any longer. When this occurred, the CR-10 score was 7.93 (±0.55). This level was higher than “very strong” (CR-10 = 7). The time when the participants stopped pushing was marked as the 0 min of fatigue recovery. The *MS* and CR-10 values measured every minute after were the muscle fatigue recovery times. The ANOVA results showed significant differences in each *MS* (*p* < 0.0001) and CR-10 (*p* < 0.0001) among the points of time during the 0–6 min period. The results of the Bonferroni post hoc test are shown in Table 4. The *MS* at the 6th min was significantly larger than those at the 0, 1st, and 2nd min. The *MS* at the 4th and 5th min was significantly larger than those at the 0 and 1st min. The *MS* at the 3rd min was significantly larger than those at 0 min. The *MS* at the 2nd min was significantly larger than that at 0 min. The CR-10 at the beginning (0 min) of a break was the highest and it decreased sequentially along the time axis indicating the progress of recovery. Pearson’s correlation coefficient between the time of recovery and *MS* (*r* = 0.98, *p* < 0.0001) and between the time of recovery and CR-10 score (*r* = −0.92, *p* < 0.01) were both high. The CR-10 score was negatively correlated with *MS* (*r* = −0.98, *p* < 0.0001).

The *MS* model for muscle fatigue recovery was constructed based on the force exertion of a body part, such as low back [27], shoulder [50], and hand [51]. The theoretical model is, on the other hand, based on the change in *MS,* such as the model of Ma et al. [23]. The model of Ma et al. [23] was selected as the basis to construct a muscle fatigue recovery model for our manual demolition tasks (see Equation (8)).

*MS* = *MS*_0_ + (*MVC*−*MS*_0_) (1−e^−*RR* × *t*^) (8)

In Equation (8), the *MS* is the *MVC* at time *t*. *MS*_0_ is the *MS* at the beginning of recovery (0 min). *RR* is muscle fatigue recovery rate, min^−1^ and *t* is time (min). *MVC* is the maximum strength before the trial started. 

By the logarithmic transformation of Equation (8), the following Equation was obtained:

(9) $$RR\times \;t=Ln\;(\frac{MVC-MS_{0}}{MVC-MS})$$

The *RR* values can be obtained by performing a linear regression analysis without an intercept using the experimental data in Equation (9) where y is equal to *L*n ((*MVC*-*MS*_0_)/(*MVC*-*MS*)) and *t* is the independent variable. The data of group A were adopted and were substituted into Equation (9) and we have Equation (10):

y = −0.132*t* (10)

The R^2^ of Equation (9) was 0.99 and the regression coefficient, or *RR*, was equal to –0.132. Substituting this *RR* value into Equation (8), we then obtained Equation (11):

*MS* = *MS*_0_ + (*MVC*−*MS*_0_)(1−e^−^^0.132*t*^) (11)

**Figure 6:**
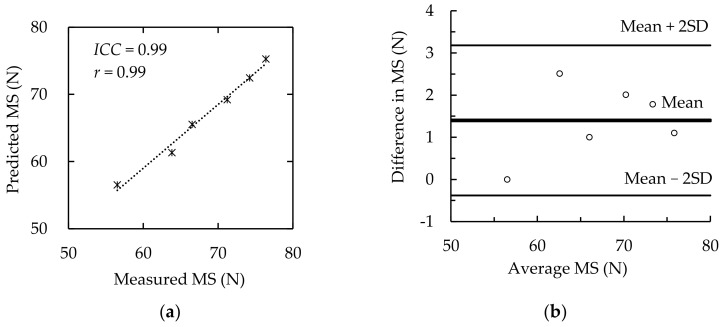
Comparison of measured and predicted *MS*. (**a**) *ICC* and *r*, (**b**) Bland–Altman agreement analysis. Difference in *MS* = Measured *MS*–Predicted *MS*; Average *MS* = (Measured *MS* + Predicted *MS*)/2.

**Figure 7:**
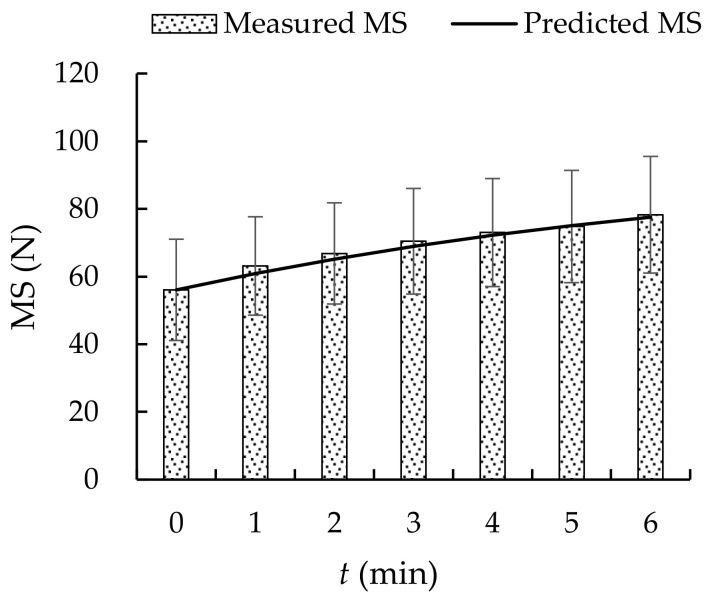
Measured and predicted *MS* showing *MS* recovery.

The AD and RD of the measured and predicted *MS*, using Equation (11) and *MS* data of group B, at 0–6 min were calculated to validate Equation (11). The AD and RD were (1.39 ± 0.81) N and (1.9 ± 1.2) %, respectively. Both the *ICC* and *r* between the measured and predicted values were 0.99 (*p* < 0.0001) (see Figure 6a). Bland–Altman analysis showed that all data were within the Mean ± 2SD interval (see Figure 6b). These data showed a fine agreement of measured and predicted *MS*. The measured and predicted *MS* calculated using Equation (11) are shown in Figure 7. The *MS* model (Equation (11)) was appropriate for describing the change in *MS* over the rest period.

## 4. Discussion 

Both in the muscle fatigue and recovery experiment, a single continuous operation fatigue induction is usually tested instead of the operation–rest–operation method in real tasks [23,24]. For manual demolition tasks, two issues should be noticed. The first was at what fatigue level the participant started the fatigue recovery test. In the literature [23,51,52], a decline of *MS* was used to indicate the development of fatigue. In their experiment, the participant was requested to exert a force until a 50% or 30% *MVC* decrease was observed. Those decreases indicated the fatigue level. In our study, the participants stopped pushing when they were exhausted. The *MS* of the fatigue recovery test at 0 min was (58.4 ± 10.1) % *MVC*. The *MS* decline was, therefore, 41.6% *MVC*, which was between the 30 to 50% range reported in the literature [23,51,52]. The second issue was the time required to observe muscle fatigue recovery. Duong et al. [24] suggested the measurement of all or at least the main information at each stage of fatigue recovery so as to better describe the progress of recovery. Although manual demolition tasks require the joint participation of all body muscles, WMSDs occur mainly in the upper extremity [53]. A recovery time of 6 min was adopted in the current study. This was consistent with those in the literature [23,36,37].

### 4.1. Muscle Fatigue 

In the muscle fatigue experiment, the load significantly affected MET (*p* < 0.0001). This was consistent with the findings in the literature [12,19,33]. It was anticipated that the weight of the tool could have an effect on the MET. The reason was that pushing a heavier tool may require additional effort to support the weight of the tool as compared to a lighter one. However, our results showed that the effect of tool weight on muscle fatigue development was not significant (*p* > 0.05). The CR-10_0_ of the small tool (8.06 ± 0.55) was significantly (*p* < 0.05) higher than that of the large one (7.79 ± 0.54). This implies that using a small hammer involved higher forceful exertion. The MET-*f_MVC_* relationship in our power predictive model (see Figure 4) was in good agreement with the models in the literature [12,16]. The general model in the literature [12,16], on the other hand, had better fitness than those upper extremity models (see Table 2). This implies that the force exertion for manual demolition tasks requires not only the effort of the upper extremity but also the joint efforts of all body parts. This might be the reason why the *MS* only recovered to 82.1% *MVC* after 6 min recovery and did not reach 95% *MVC* of the *MS* as suggested in the literature [17,40]. 

### 4.2. Muscle Fatigue Recovery 

By observing the *MS* data at each time point, it was found that the mean *MS* values at 0, 1, 2, 3, 4, 5, and 6 min were 58.8%, 66.2%, 70.1%, 73.9%, 76.6%, 78.4%, and 82.1% *MVC*, respectively. After having a rest of 6 min, the *MS* did not recover to 95% of the *MVC* [17,40]. According to our *MS* prediction model (see Equation (10)), it will take approximately 16 min to recover 95% of the *MVC* in our tasks. Although the general models in the literature (see Table 2) could fit the data of our demolition tasks better than those of the upper limb models, they were constructed without considering the force applied. The recovery model established in the current study is applicable in assessing manual demolition tasks. 

**Figure 8:**
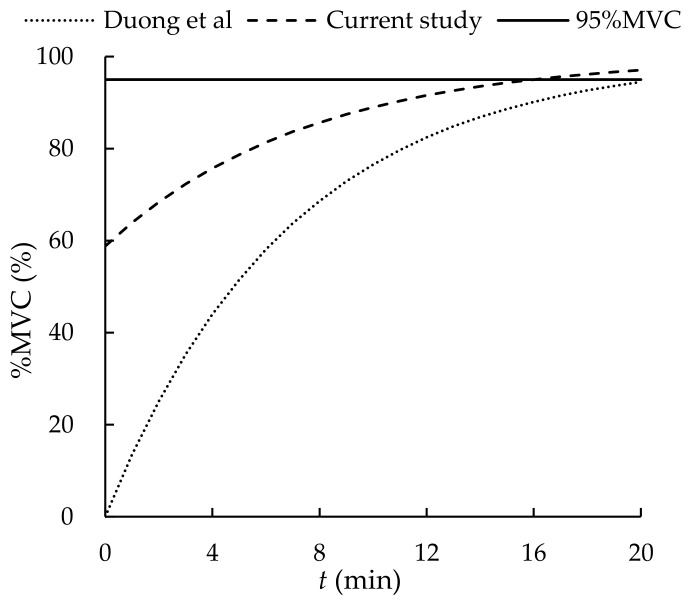
Recovery of muscle fatigue.

Duong et al. [24] arranged a break of 20 min for their participants and recorded 0% to 95% recovery of their *MVC*. Fulco et al. [51] adopted a break of 3 min and found 53% to 70% and 51.9% to 73% *MVC* recovery for their male and female participants, respectively. Yassierli et al. [50] reported 95% *MVC* recovery after a break of 15 min. In the current study, the break time was 6 min and the *MVC* recovery was between 58.8% and 82.1%. According to Equation (11), it will take approximately 16 min to reach a 95% *MVC* recovery. The recovery rate in Duong et al. [24] seems to be faster than that of ours (see Figure 8). This could be attributed to the fact that the posture and *MS* in Duong et al. [24] were different from those in our study. Nevertheless, an obvious faster recovery can be observed in 0–6 min in both Duong et al. [24] and ours (see Figure 8). This might be attributed to the recovery pattern of energy reserves after performing forceful tasks. After an exhaustive exercise, the recovery of oxygenated myoglobin is fast. It takes only about 1 min for a full recovery [54]. The full recovery of phosphagen (ATP, PC) in the muscle needs approximately 2 min, but the consumed phosphate may be synthesized within 20–30 s [54]. These may explain the faster increase in *MS* during the early phase of the muscle fatigue recovery test in the current study.

**Figure 9:**
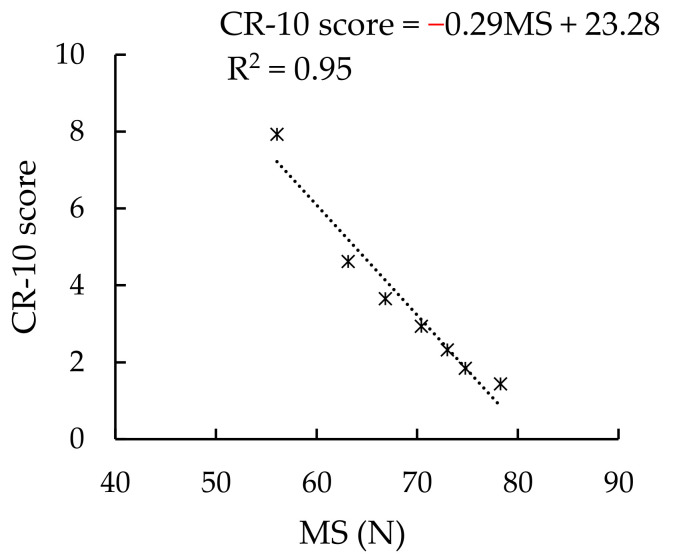
CR-10 score-*MS* relationship.

In the fatigue recovery test, the difference in the CR-10 score among 0–6 min time periods were significant (*p* < 0.0001), and CR-10 was negatively correlated with *MS* (*r =* −0.98, *p* < 0.0001). Figure 9 shows the relationship between the *MS* and CR-10 score. The decrease in the CR-10 score along with the increase in the *MS* clearly indicated the recovery of the muscle. 

The *MS* prediction model was established based on the theoretical model of Ma et al. [23]. Validation was carried out for this *MS* model. The *ICC* and *r* values and Bland–Altman results for the predicted and measured *MS* values indicated that our *MS* model is appropriate in describing the fatigue recovery phenomenon of our manual demolition tasks.

### 4.3. Limitations 

This research has limitations. First, only two hammers were tested. Some of the hammers used by professional demolition workers are much larger and heavier than those in our study. Our results and models may not be applied to those hammers. Second, our participants were novices without prior experience in using a demolition hammer. Tool handling skills between novices and experienced workers are different. Our results, therefore, can only be applied to novice workers. Third, the power of our hammers was not turned on because of safety considerations. There were no vibrations, such as those in real hammering tasks. Our results could be different if there were vibrations in the tasks. In addition, only one posture was tested. The demolition target was at the average elbow height of the Chinese male population [39]. In real demolition tasks, the height of the demolition target may be at any level other than the one tested in the current study. The target height affects the posture of the worker and hence, the development of muscle fatigue. Different target heights may be tested in the future to determine the effects of target height on the development and recovery of muscle fatigue. Finally, the literature shows that different intervention measures affect fatigue recovery [55,56]. In the future, studies exploring the development of muscle fatigue recovery under different intervention measures may be conducted to reduce the accumulation of muscle fatigue and thus reduce the risk of WMSDs.

## 5. Conclusions

In manual demolition tasks, push force significantly affected the MET and hence, the development of muscle fatigue. The effects of the weight of the tool were not significant. The MET predictive model developed in the current provided better fitness on the MET data than the models in the literature. For the progress of *MS* recovery, the *MS* model developed, based on a theoretical model in the literature [23], provided reasonable estimates of the change in muscular strength under different rest times for manual demolition tasks. Future research is required to explore the issues of manual demolition tasks mentioned in Section 4.3 to fill the gaps of our understanding on both the development of muscle fatigue and recovery for manual demolition tasks.

## Figures and Tables

**Table 1:** MET values under experimental conditions.

Push Force (N)	Hammer	MET (min)
20	small	6.93 (±3.11)
	big	7.93 (±3.70)
40	small	2.45 (±0.63)
	big	2.41 (±0.95)

**Table 2:** MET models for muscle fatigue in the manual demolition tasks.

Function Form	Regression Equation		R^2^	*p*
Exponential functions	y = −4.813x + 2.879	(a)	0.69	*p* < 0.0001
	y = 3.392x	(b)	0.56	*p* < 0.0001
Power functions	y = −1.548x − 0.516	(c)	0.72	*p* < 0.0001
	y = −1.15x	(d)	0.94	*p* < 0.0001

**Table 3:** AD and RD values of the predictive models.

Models		AD (min)	RD (%)
General Model	Sjogaard [43]	2.04 (±2.22)	48.01 (±47.70)
	Rose et al. [44]	2.93 (±2.94)	48.65 (±18.30)
Upper limb model	Sato et al. [45]	3.06 (±3.02)	50.72 (±19.95)
	Mathiassen and Ahsberg [46]	2.51 (±1.88)	55.70 (±27.19)
Back/hip model	Manenica [47]	2.85 (±2.73)	82.19 (±91.30)
Current study	Group A data	1.32 (±1.69)	30.49 (±27.22)
	Group B data	1.83 (±1.94)	34.80 (±31.48)

Note: AD: absolute deviation; RD: relative deviation.

**Table 4:** Bonferroni post hoc test results of *MS* and CR-10 score.

Time (min)	*MS* (N)	CR-10 Score
0	56.06 (±14.97) ^A^	7.93 (±0.55) ^A^
1	63.13 (±14.54) ^AB^	4.62 (±1.44) ^B^
2	66.84 (±14.95) ^BC^	3.65 (±1.28) ^C^
3	70.42 (±15.61) ^BCD^	2.93 (±1.10) ^D^
4	73.00 (±15.96) ^CD^	2.32 (±0.85) ^E^
5	74.79 (±16.59) ^CD^	1.84 (±0.84) ^EF^
6	78.27 (±17.23) ^D^	1.43 (±0.72) ^F^

Note: Numbers in the parentheses are standard deviation; different letters indicate that they are statistically different; α = 0.05.

## Data Availability

Data available upon request.
